# AGING-FRIENDLY COMMUNITY INITIATIVES: HARNESSING NETWORKS FOR CHANGE FROM THE LOCAL TO THE GLOBAL

**DOI:** 10.1093/geroni/igz038.1979

**Published:** 2019-11-08

**Authors:** Emily Greenfield, Laurent Reyes

**Affiliations:** 1 Rutgers University, New Brunswick, New Jersey, United States; 2 Rutgers, The State University Of New Jersey, New Brunswick, New Jersey, United States

## Abstract

Aging-friendly community initiatives (AFCIs) represent multi-sectorial efforts to systematically make localities better for long lives. AFCIs typically work toward progress in multiple domains of community life, focusing both on social and built environments. Despite tremendous growth in AFCIs worldwide over the past decade, there has been little empirical research on how they develop and work toward their aspirational goals. This paper presents findings from a community-partnered study on the development of nine philanthropically supported AFCIs in northern New Jersey. Drawing on fives waves of in-depth interview data collected with leaders over three years, the paper addresses the role of the leaders’ interpersonal and interorganizational networks as they work toward aging-friendly community change. Findings on the centrality of networks across various levels of geographic scale for the development of AFCIs has implications for program theory, as well as broader issues concerning inclusion and equity within community-level responses to population aging.

